# Patching Up the Permeability: The Role of Stem Cells in Lessening Neurovascular Damage in Amyotrophic Lateral Sclerosis

**DOI:** 10.1093/stcltm/szac072

**Published:** 2022-10-01

**Authors:** Molly Monsour, Svitlana Garbuzova-Davis, Cesario V Borlongan

**Affiliations:** Morsani College of Medicine, University of South Florida, Tampa, FL, USA; Center of Excellence for Aging and Brain Repair, Morsani College of Medicine, University of South Florida, Tampa, FL, USA; Center of Excellence for Aging and Brain Repair, Morsani College of Medicine, University of South Florida, Tampa, FL, USA

**Keywords:** neurovascular, amyotrophic lateral sclerosis, stem cells, blood central nervous system barrier, permeability

## Abstract

Amyotrophic lateral sclerosis (ALS) is a debilitating disease with poor prognosis. The pathophysiology of ALS is commonly debated, with theories involving inflammation, glutamate excitotoxity, oxidative stress, mitochondria malfunction, neurofilament accumulation, inadequate nutrients or growth factors, and changes in glial support predominating. These underlying pathological mechanisms, however, act together to weaken the blood brain barrier and blood spinal cord barrier, collectively considered as the blood central nervous system barrier (BCNSB). Altering the impermeability of the BCNSB impairs the neurovascular unit, or interdependent relationship between the brain and advances the concept that ALS is has a significant neurovascular component contributing to its degenerative presentation. This unique categorization of ALS opens a variety of treatment options targeting the reestablishment of BCNSB integrity. This review will critically assess the evidence implicating the significant neurovascular components of ALS pathophysiology, while also offering an in-depth discussion regarding the use of stem cells to repair these pathological changes within the neurovascular unit.

## Teaser

This review article elucidates the role of the neurovascular unit in Amyotrophic Lateral Sclerosis and discusses the potential role of stem cells in targeting this underlying pathophysiology.

## Graphical Abstract

**Graphical Abstract:**
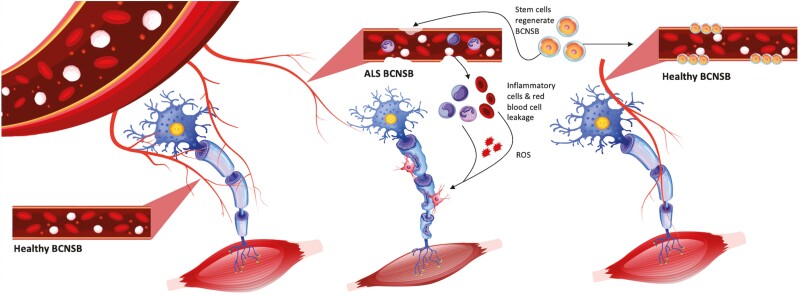

**Significance Statement**

This review elucidates the role of the neurovascular unit in amyotrophic lateral sclerosis and discusses the potential role of stem cells in targeting this underlying pathophysiology.

## Background

Amyotrophic lateral sclerosis (ALS) is an incredibly detrimental disease characterized by motor neuron (MN) degeneration and subsequent muscle atrophy, weakness, fasciculations, and paralysis.^1^ ALS is diagnosed in 1-2.6 per 100,000 individuals each year and has an overall prevalence of 6 per 100,000 people, with most dying 3 to 5 years after diagnosis.^2-4^ This short life span is largely due to the disease’s complex underlying mechanisms complicating treatment development. Currently, only Riluzole, a glutamate signaling blocker, and Edavarone, a reactive oxidative species (ROS) mitigator, are FDA-approved ALS pharmaceuticals.^5,6^ To advance treatment development, the pathophysiology of ALS must be more substantially uncovered. Most cases of ALS are sporadic (90%), but some patients have genetic predispositions from gene mutations in *SOD1*, *TDP-43*, or *FUS*.^7,8^ In addition to these genetic contributions, theories of motor neuron degeneration center around glutamate excitotoxity, oxidative stress, mitochondria malfunction, neurofilament accumulation, inadequate nutrients or growth factors, changes in glial support, and inflammation.^9-16^ While all these theories are supported individually, they also seem to result in or arise from a common factor: neurovascular disruption.^11,17^ This review will focus on the overwhelming support of for neurovascular changes contributing to ALS pathophysiology, and prompt further study of the use of stem cells (SC) to restore and strengthen the neurovascular damage propagating ALS symptoms.

## An Unknown Pathology or a Cascade of Consequences?

### Vast Possibilities: Putting the Blood Vessel Under the Scope

The origins of ALS, particularly in the more common sporadic cases, continue to be a medical mystery. The hypothesis that hypoxia induces motor neuron damage becomes evident when deleting the hypoxia response element in mice, which ultimately inhibits vascular endothelial growth factor (VEGF) and produces an ALS-like mouse model.^18^ Hypoxia may also induce an inflammatory response, further diminishing the motor neurons’ viability.^19^ Lymphocytes, pro-inflammatory cytokines (IL- 1α, IL-1β, TNF-α, and IL-1RA), pro-inflammatory enzymes (COX-2), immune response elements such as IgG, and activated immune cells are frequently elevated in ALS patients’ spinal cords.^11,20-24^ In addition to the inflammatory cytokines released by activated immune cells, reactive oxidative species (ROS) are released and elevated in the cerebrospinal fluid (CSF), blood, and urine of patients with ALS, while antioxidants such as reduced glutathione (GSH) and nitric oxide (NO) are decreased in the plasma.^25-28^ Mitochondrial function, also important in regulating ROS production, shows altered functionality in skeletal muscle tissue of ALS mice models.^29^ Each of these factors may propagate cell death, leading to extreme amounts of glutamate release and injurious excitotoxicity.^30^ Transgenic mice with downregulated membralin, a protein responsible for modulating EAAT2 activity, develop into an ALS-like model. EAAT2 rids the synaptic clefts of glutamate and reduces excitotoxicity. Clinical measures of membralin and EAAT2 in the spinal cord are also abnormal in ALS patients. Upregulating membralin, however, aptly improved MN viability and increased EAAT2 expression.^31^ This wide range of clinical and preclinical therapeutic targets overwhelms effective treatment development; however, the multitude of changes seen in ALS converge on common disruption of the neurovascular unit (NVU).^11^

### The Neurovascular Unit

The NVU was first described in 2001 as a cohesive, interdependent relationship between the brain and surrounding vasculature.^32^ A high-energy–consuming organ, the brain requires continuous glucose and oxygen delivery, a primary role of the NVU.^33^ Furthermore, this imperative relationship allows for successful brain development, adult neurogenesis, and neuronal regeneration.^34-36^ At its basis, the NVU is mainly composed of neurons, glial cells, endothelial cells (EC), vascular smooth muscle cells (VSMC), pericytes, and a basement membrane including collagen IV. These components promote a homeostatic environment for the brain.^37,38^ Astrocytes, a predominant glial cell population in the NVU, have feet projections which wrap around 95% of the blood vessels and enhance the blood brain barrier (BBB) and blood spinal cord barrier (BSCB) impermeabilities.^11,39,40^ These feet processes are further stabilized by intertwined pericytes, which receive signals, such as platelet-derived growth factor (PDGF) and transforming growth factor-β (TGF-β), from ECs to modulate vessels in the NVU.^39,41^ Pericytes are also vital to BBB impermeability and primarily work to remodel and stabilize the microvasculature within the NVU, while also contributing to immune and inflammatory responses.^41^ Local microglia and macrophages within the perivascular space also contribute to immune and inflammatory responses by regulating immune and inflammatory cell populations in the central nervous system (CNS) and removing unwanted materials.^42,43^ Yet another stabilizing function of astrocytes includes their role in tight junction formation between endothelial cells and transporter protein migration to the surface.^44-46^ These tight and adherens junctions, prevalent between endothelial cells of the NVU and vital to BBB and BSCB integrity, are made of various proteins, including cadherins, occludins, zonula occledins (ZO), junctional adhesion molecules, and claudins.^47,48^ The transporters within the NVU, such as breast cancer resistance protein and P-glycoprotein, maintain homeostasis by modulating what can enter and leave the brain based on the local environment.^11^ EC function is further modulated by vascular smooth muscle cells (VSMCs). These cells control pressure in the veins, arteries, and arterioles, while also controlling endothelin-1 and angiotensin II receptor proliferation in the vessels. Once again, astrocytes also play a defining role in this function by releasing endothelin-1 and angiotensin II.^38^ Overall, each of these cells play a large role in vasoconstriction within the NVU.^38,49^ Given the NVU’s vital role in maintaining CNS homeostasis through the BBB and BSCB, collectively considered as the blood central nervous system barrier (BCNSB), it is understandable that the ALS-induced inflammatory mediators and ROS impacting the NVU can be incredibly detrimental.

## ALS with Neurovascular Components

The defined inflammatory, oxidative, excitotoxic, and hypoxic conditions observed in ALS contribute to BBB and BSCB damage in unique ways, disrupting homeostasis and ultimately leading to MN damage and death.^11^ Microglia are reported in the spinal cords of animal models of ALS, later releasing pro-inflammatory cytokines and recruiting inflammatory monocytes even before symptom progression.^50,51^ Microglia also release IL-1α, TNF-α, and C1q to recruit astrocytes.^52,53^ These activated astrocytes further worsen motor neuron degeneration in ALS and impair cell migration, angiogenesis, and repair.^11,54^ Thus, inflammation induced by activated microglia and astrocytes may play a prominent role in BCNSB disturbance in ALS. However, some research supports BCNSB disruption precedes the toxic environment seen in ALS. This early barrier disruption allows for easy accumulation of pro-inflammatory immune cell invasion.^19^ Regardless of the progression of pathological NVU changes, ALS is accompanied by EC, astrocyte feet processes, tight junction, and vascular integrity disruptions.^11,55^ These disturbances allow for microhemorrhages, hemosiderin deposits, and other external substances to enter the typically isolated CNS environment, stimulating ALS disease progression.^11,56^ The subsequent sections offer preclinical and clinical evidence implicating the key involvement of impaired neurovasculature in ALS.

### Preclinical Evidence of NVU

Various preclinical studies using different murine models or cell cultures of ALS have demonstrated BBB and/or BSCB permeability, ultimately contributing to ALS pathology. In G93A SOD1 ALS mice models, a common model of ALS, Evans blue leakage is noted in spinal cord capillaries incredibly early in disease progression.^51^ This leakage signifies BCNSB permeability. IgG and a polar ionic tracer, rubidium-86 chloride, can also surpass the BSCB in ALS mice models.^57^ Downregulated Glut-1, CD146, and laminin are also prominent in ALS mice, signifying endothelial and basement membrane damage.^58^ Further evidence for vessel disruption in this mouse model includes degenerated ECs, astrocytes, extracellular edema, mitochondrial degeneration in ECs, and astrocyte end feet swelling surrounding NVU capillaries visualized through electron microscopy. Erythrocytes are also observed in the spinal cord, substantiating BCNSB insufficiency and leakage at early ALS stages.^59^ Edema may be due to altered aquaporin 4 expression in astrocyte end feet, allowing for excess water accumulation.^48,60^ Reducing aquaporin 4 expression in ALS mice improves the BBB integrity but impairs the animals’ time to disease onset and lifespan.^61^ Other researchers have supported findings on weakened BCNSB in ALS, noting changes such as decreased platelet-endothelium cell adhesion molecule (PECAM-1), occludin, and collagen IV from the basement membran0e in spinal cord tissues in ALS mice.^62^ BCNSB damage may even precede disease onset, making it a vital aspect of pathogenesis to consider in treatment development. ZO-1, occludin, claudin-5, and Glut-1 are decreased prior to symptom onset in G93A SOD1 mice. Also before disease onset in the ALS mice, 30%-45% of blood flow is reduced in lumbar and cervical spinal cords and capillary lengths are reduced by 10%-15%. Microhemorrhages and hemosiderin deposits observed in the spinal cord support findings insinuating BCNSB weakening prior to symptom onset.^19^ Similar findings in ALS mice have noted thickened basement membranes, increased pericytes, reduced capillary, and tight junction densities prior to NMJ denervation, suggesting that early NVU changes precede ALS symptoms.^63^ Each of these findings supports significant contributions from faulty NVU integrity in ALS pathophysiology.

While BCNSB permeability is apparent in these preclinical models, elucidation of the causes of this permeability may guide the development of novel therapeutics. In vitro studies of ECs show astrocytic, microglial, neuronal, and pericyte roles in BCNSB integrity. Similarly, inflammatory cells, such as neutrophils, lymphocytes, and monocytes, can alter barrier permeability.^11,48,64-68^ ECs, neurons, astrocytes, and microglia have the potential to release matrix metalloproteinases (MMPs), which break down extracellular matrix components such as laminin, fibronectin, proteoglycans, and collagen IV.^69-71^ MMP-9 and MMP-2 are elevated in mouse models of ALS, suggesting this extracellular matrix breakdown contributes to NVU disruption.^72^ Prior to MN death, MMP-9 is seen in large motor neurons, microglia, and endothelial cells, leading to astrocyte end feet damage and capillary leakage in ALS mice.^62^ MMP-9 elevations and decreased collagen IV are also localized to the anterior half of the lumbar cord, an area rich in MN, and blood vessels in ALS mice, further supporting this protein’s contributions to ALS pathology via NVU disruption.^62^ In addition to MMP damage, activated microglial release of IL-1α, TNFα, and C1q can induce a pro-inflammatory differentiation of astrocytes. This differentiation promotes neuronal damage and knocking out IL-1α, TNFα, and C1q signals in mice models of ALS elongates their life spans.^52,53^ Inflammatory responses also detriment tight junction function, further contributing to BCNSB weakening. In rats, TNF-α, IFN-γ, and TGF-β reduce the expression of tight junction proteins such as occludin, claudin, and ZO-1.^73^ TNF-α, IFN-γ, and TGF-β are increased in ALS, encouraging BCNSB alterations.^74,75^ Concurrent with inflammation, another hypothesized contributor to ALS pathology is TDP-43 accumulation. It is theorized that ALS induced inflammation promotes TDP-43 detrimental effects. Overexpressing TDP-43 in mice with E. coli-induced systemic inflammation shows exacerbated IgG, CD3, and CD4+ T-cell accumulation in the CNS, activation of ECs and pericytes, impaired BBB stability, neuronal death, and impaired functionality.^76^ In conditional knockout mice with TDP-43 not expressed in motor neurons, endothelial swelling and vacuolization is noted; however, tight junctions are not disrupted, pericyte damage is not observed, and the BSCB is preserved at later stages of ALS progression.^77^ Other studies suggest BSCB leakage and TDP-43 proteinopathy are independent, as high hemoglobin concentrations, signifying BSCB permeability, do not overlap with high TDP-43 areas in post-mortem studies.^78^ These results encourage further investigation of the role of TDP-43 in ALS NVU disruption. While inflammatory responses can worsen BCNSB permeability, there may be a bidirectional pathway between inflammation and barrier damage in ALS, as BCNSB damage has been observed prior to any elevated inflammatory markers in mice models.^19^ The communication between inflammatory mediators and the BCNSB in ALS must be further clarified to design optimal treatments to reduce this pathogenic contributor in ALS symptoms.

Alongside inflammation, ROS accumulation substantially damages MNs and weakens the BCNSB in ALS.^79^ In vitro blood brain barriers developed from SOD1-G93A ALS models show increased ROS production, Nrf2, and NF-kB activation in endothelial cells due to mutant astrocyte signaling.^80^ In rats, pre-ALS and ALS brains have elevated nitrilation, superoxide production, lipid peroxidation, and manganese superoxide dismutase activity, all markers of elevated oxidative stress. Copper-zinc superoxide dismutase activity, encoded by SOD1, is expectedly decreased in the SOD1 G93A rat model.^81^ SOD1, a known contributor to familial ALS, is involved in oxidative resistance and repair gene transcription.^82^ Thus, when damaged, it is understandable that ROS have been a major area of focus for understanding ALS pathology. An in vitro model of human umbilical cord-derived endothelial vascular cells (HUVEC) and astrocytes treated with ALS plasma shows elevated mitochondrial ROS production.^83^ ROS promote phosphorylation and ubiquination of tight junction occludin proteins, marking them for degradation and weakening the BCNSB.^84^ Like inflammation, oxidative stress may induce or be induced by BCNSB damage. Due to a weakened BSCB, hemoglobin and iron infiltration in the spinal cord amplify MN degeneration in ALS mice through oxidative stress. These effects are further exemplified by administering warfarin, an anti-coagulant, to accelerate lesion formation. By increasing these toxic blood components and oxidative stress, MN damage was accelerated. Contrarily, activated protein C administration to block oxidative damage from warfarin induced microvascular lesions mitigates this toxic effect, slowing MN damage and functional deficits.^85^ While designing BCNSB targeted therapies for ALS, this bidirectional communication between ROS and the barriers of the NVU must be considered.

Hypoxic conditions may induce these inflammatory and oxidative conditions in ALS, ultimately disrupting the NVU. In spinal cords of rat ALS models and brains of ALS patients, blood flow is notably disrupted which likely impairs oxygen and glucose delivery within the NVU and establishes a hypoxic environment.^19,86^ An intriguing theory involving the NVU and ALS suggests that hemodynamic changes contribute to altered BCNSB integrity and instigate hypoperfusion of the CNS. Pathological hemodynamics initiate venous constriction, leading to high venous pressure and capillary distension. Capillary distension allows for forced tight junction separation, subsequent vessel leakage, and ultimately inadequate blood flow.^87^ This theory is supported by upregulated endothelin-1, a vital protein for venous constriction, in ALS mouse models’ spinal cords, provoking these hypoxic conditions.^88^ Also demonstrating the role of hypoxia in ALS, inhibiting the hypoxia response element in the *VEGF* gene promotor blocks neovascularization in the face of hypoxia and can lead to MN death.^79^ In fact, Riluzole, one of the few marketed ALS treatments, induce neuroprotective via increased hypoxia resistance in human umbilical vein endothelial cell and bovine retinal endothelial cell cultures and rats.^89^ Considering the BCNSB, hypoxia and subsequent astrocyte production of hypoxia inducible factor (HIF)-alpha and VEGF-A reduce claudin expression in mouse models of multiple sclerosis. Similar hypoxic conditions seen in ALS may result in diminished tight junction proteins impaired BCNSB.^90,91^ Thus, restoration of oxygen and nutrient delivery through restored vessel integrity should be a primary target of ALS therapeutic development.

As a result of these accumulated pathophysiological mediators, impaired BCNSB integrity can lead to glutamate excitotoxicity. Barriers of the CNS contain excitatory amino acid transports (EAAT) to remove glutamate and maintain homeostatic levels.^92,93^ When these barriers are insufficient, however, glutamate removal is also impaired, and excitotoxic effects are eminent. To mitigate this damage, astrocytes signal to the endothelial cells to upregulate the multidrug resistance transporter ABCB1 (P-Glycoprotein) (P-gp), which can also modulate glutamate levels.^94,95^ While vital to reducing excitotoxicity, the effectiveness of Riluzole is limited by the upregulation of the P-gp seen in ALS.^80,94,96^ Appropriate P-gp levels are necessary to achieve therapeutic benefits of most pharmaceuticals,^97^ thus, the BCNSB integrity should be a primary target for all future therapies for ALS. Without reimplementing this barrier and normalizing P-gp expression, other pharmaceutical interventions would be futile.

Given the vast array of reports on NVU disruption in ALS, diagnostic and therapeutic advancements must be made to efficiently reduce BCNSB permeability. X-ray phase contrast tomography (XPCT 3D) may be a promising tool to quickly diagnose ALS, as it shows vascular disruptions in ALS mice spinal cords at pre-symptomatic stages.^98^ In rat models, Electron paramagnetic resonance (EPR) spectroscopy can measure BBB permeability and redox status, another promising initiative for early BCNSB damage intervention.^81^ T2*-weighted MRI and Gd-DTPA may also be helpful tools for measuring BCNSB permeability, as they have both been used in rat models to demonstrate barrier weakening and NVU alterations.^24,99,100^ By discovering BCNSB permeability as early as possible in patients with ALS, rapid intervention can be achieved, and positive prognoses may be more attainable than ever before.

### Clinical Manifestations of Compromised NVU in ALS

While animal models of ALS offer incredible insight into the pathophysiology of the disease, ALS has an incredibly complicated molecular basis and difficult to mimic at a preclinical level. Most ALS models involve transgenic animals, despite the majority of ALS cases being sporadic. Thus, it is important to study BCNSB strength in a clinical setting to confirm whether it is equally permeable without contributing familial genetic mutations. Early accounts of elevated IgG, albumin, and complement proteins in the spinal cords and brains of patients with ALS instigated theories regarding the role of BCNSB permeability in MN death.^101-104^ More recently, reports of endothelial damage, pericyte degeneration, intra- and extra-cellular edema, reduced ECs, abnormal levels of collagen IV, increased microvascular density in the spinal cord, leakage products from permeable BCNSB, and reduced EC junction proteins support severe BCNSB fragility in patients with ALS.^105^ Post-mortem studies of patients with ALS have revealed 54% reduction in pericytes within the spinal cord, signifying immense BSCB damage.^106^ In surviving patients, platelet derived growth factor (PDGF) levels are increased in an effort to restore pericytes in the BCNSB.^107^ Tight junction destruction is another clinical observation transferred from pre-clinical discoveries. mRNA of ZO-1 and occludin are decreased in sporadic and familial cases of ALS.^101^ The NVU is further impaired by misplaced astrocytic end feet and decreased basement membrane formation due to reduced collagen IV.^62,108^ Basement membrane damage due to reduced collagen can be seen directly in the ventral horns of the spinal cord, exemplifying its profound role in MN damage and ALS pathology.^109^ BCNSB permeability is clearly recognized preclinically as well as clinically, but further unveiling of the pathophysiology is needed to develop effective treatments.

This immense damage to the NVU and corresponding permeability may be due to inflammation, oxidative signals, or hypoxia, similar to the factors seen preclinically. For instance, inflammatory mediators and MMP-9 elevations are noted in the serum and in spinal cord tissues of patients with ALS.^62,110-112^ Elevations in MMP are observed outside of the CNS as well, further damaging surrounding tissues in ALS.^113^ Also like animal models, endothelin-1 expression is upregulated in spinal cords of patients, suggesting a similar pathological venous reflux and subsequent tight junction separation in ECs.^88^ Increased endothelin-1 may be due to hemodynamic instability seen in ALS patients.^114^ Patients with ALS have a significantly increased mean transit time for perfusion of all cortical regions and impaired microcirculation.^114,115^ These hemodynamic changes and subsequent hypoxia may prelude the enlarged perivascular spaces seen in patients’ spinal cords. To restore blood flow, endothelin-1 may be overexpressed, ultimately separating astrocytes from the basement membrane. This is further supported by perivascular fibroblast infiltration in the BSCB, which may be an attempt to restore barrier integrity.^67,87,88,116^ Elevated oxidative damage is also observed. In a sample of 25 patients, the plasma showed lipid peroxidation elevation and decreased anti-oxidants such as nitric oxide and reduced glutathione.^83^ This oxidative damage may arise from erythrocyte infiltration into the CNS through gaps in the NVU barriers. Erythrocytes possess iron-containing hemoglobin and hemosiderin, both of which can induce oxidative stress through the Fenton reaction.^117^ Using MRI, reports of increased iron in microglia in the motor cortex may signify vascular leakage due to BCNSB permeability in ALS.^118^ In a study of 11 patients with ALS, a 3.1 fold increase in perivascular hemoglobin deposits compared to controls was observed in the vascular lumen. These patients also had increased IgG, fibrin, and thrombin in the spinal cord, however, pericyte number was decreased by 54% compared to controls. Pericyte number was negatively correlated to BSCB damage and hemoglobin concentrations, supporting the role of BCNSB damage in oxidative stress.^106^ There is a plethora of well conducted studies encouraging extensive research on BCNSB restoration in ALS to delay disease progression, reduce symptoms, and prolong lifespans.

## Restoring the NVU

Underlying BCNSB permeability in ALS is a wide array of cellular damage and dysfunction. Thus, it is logical to develop treatments aimed at restoring these faulty cells, eventually recovering BCNSB integrity. Stem cells have recently revolutionized a wide variety of neurological diseases, including ALS.^119^ Currently, the treatment for ALS is focused on palliative care, with only two FDA approved and moderately effective drugs available, Riluzole and Edaravone.^5,6,120-122^ These limited treatment options promote further study of cell-based treatments for ALS. Intraspinal administration of hematopoietic mesenchymal stem cells (hMSCs) in a mouse model of ALS survive and enhance functionality and MN viability, potentially via increased glial cell line-derived NTF (GDNF) expression.^123^ Human neural stem cells (NSCs) in SOD-1 transgenic rat models of ALS may also act via GDNF and brain-derived NTF (BDNF), as NSCs implanted into the lumbar spine of rat models demonstrates increased BDNF and GDNF, delays motor disease onset, and elongates the ALS rat lifespan.^124^ BDNF alone has also showed therapeutic potential^125,126^; however, NSCs offer further benefit through differentiation into neurons, which proceed to form axons and synaptic connections with endogenous MNs. Intriguingly, these NSCs could be influenced by their environments to take a neuronal or astrocytic differentiation pathway.^127^ These findings prompt future studies to prompt NSCs to adopt astroglia phenotypes and restore NVU strength. If administered early, these new astrocytes may be able to lessen the impending damage resulting from NVU permeability. Given alone, SCs demonstrate hopeful preclinical results for ALS treatment.

Given the promising results of NTF proliferation after SC administration, other studies have reinforced these neuroprotective properties by overexpressing NTFs in SCs. In mouse models of ALS, muscle progenitor cells (MPC) expressing various neurotrophic factors (NTF), such as BDNF, GDNF, VEGF, or insulin-like growth factor-1 (IGF-1), demonstrate longer times until disease onset and death, and improved neuromuscular junction (NMJ) and axon stability.^128^ Similarly, in hMSCs, hMSC overexpressing GDNF or VEGF had substantial functional and survival successes in a rat model of ALS. These results were substantiated ex vivo, as VEGF and GDNF demonstrate NMJ and MN protection in cell cultures.^129^ This has been supported by other studies reporting improved muscle cell viability, neuromuscular connections, and MN survival with GDNF producing hMSCs.^130^ There are some limitations, however, as GDNF producing neural progenitor cells do not prompt muscle reinnervation by the MNs, only provide neuroprotection of MNs.^131^ Additionally, there are conflicting reports of which NTFs are the most beneficial for MN protection, which NTFs achieve symptom reduction, and the overall safety of GDNF SCs.^132^ Avoiding these complications, further enhancement of stem cell viability and efficacy for ALS may involve reducing environmental excitotoxicity. AMD3100, an antagonist of the chemokine receptor CXCR4, blocks CXCL12 binding and subsequent glutamate-mediated apoptosis of glial cells. By inhibiting this signaling, apoptosis is decreased, and hematopoietic stem and progenitor cell infiltration to damaged regions is achievable. Thus, AMD3100 administration can improve cell-based approaches to ALS by prompting BSCB recovery and restoration of impermeability.^133^ Given the substantial role of NVU permeability in ALS pathology, further advancements in cell-based treatments to restore BBB and BSCB integrity are necessary.

To restore the NVU, preclinical studies have focused on cell-based therapies to restore impermeability of the BBB and BSCB. Human bone marrow CD34+ (hBM34+) cells implanted into ALS mice can ameliorate symptoms, improve MN survival, and migrate to capillaries to become ECs and restore impermeability of these vessels. These cells also restore the BCNSB by mitigating astrogliosis, microgliosis, strengthening the basement membrane, and improving astrocyte end-feet stability.^134,135^ These cells also reduce microhemorrhages in ALS mice due to improved vascular strength.^136^ While hBM34+ cells offer incredibly hopeful results for ALS treatments, they require high dosages to be effective.^134-136^ Thus, further differentiation of this cell line was hypothesized and shown to amplify their therapeutic potential. In vitro study of ALS mouse model cells show that human bone marrow endothelial progenitor cells (hBMEPC) can increase VEGF-A and angiogenin-1 levels, differentiate into ECs, and express cell membrane proteins such as occluding and ZO-1. Each of these in vitro findings encourages restoration of the BCNSB.^137^ Further application of these cells in vivo demonstrates their capabilities to infiltrate and restore capillary strength in the NVU, decrease Evans blue dye extravasation, and regenerate BCNSB impermeability via reformation of perivascular astrocyte end feet. Through each of these molecular changes, improved functionality and MN survival are also notable in this mouse model.^138^ The underlying mechanism for these cells’ benefits may be due to extracellular vesicle (EV) release, as EVs from hBM-EPCs reduce mouse brain endothelial cell damage when exposed to ALS mouse model plasma in vitro. These findings are especially pertinent given the risks of tumorigenicity, imprecise localization, and immune reactions following stem cell administration.^139^ In addition to cell type, the route of administration may be clinically relevant.

While stem cell delivery directly to the CNS or spinal cord via intrathecal injection may be beneficial to ensure adequate delivery, the less invasive nature of intravenous injection may be more attractive and minimize adverse outcomes.^140^ Furthermore, considering the increased BCNSB permeability in ALS, stem cells may not even need direct implantation within the CNS to reach these targets. In a SOD1 mouse model of ALS, intravenous human umbilical cord blood (hUCB) mononuclear cell treatment increases the life span of the mice.^141,142^ This life span improvement is dependent on stem cell dosage, as a dose of 70.2-73.3 × 10^6^ cells shows a greater positive impact than 33.2-33.4 × 10^6^ cell dosage. Interestingly, the larger cell dosage was obtained from many donors and still more successful than the smaller dose, prompting future study regarding intravenous administration and cell dosage.^141^ hUCB cells have been used in G93A mice models of ALS as well, and show delayed disease progression, increased life span, and substantial cell viability. These cells migrate to damaged motor neuron regions in the CNS and express neuronal cell markers. Notably, these cells are also found in peripheral inflammatory organs, like the spleen.^143^ The spleen has been deemed a predominant contributor to neuroinflammation in stroke^144^ and may be a novel therapeutic target for ALS as well. The positive outcomes of intravenous administration are also seen with mesenchymal stem cells (MSCs). SOD1 mice models injected with MSCs intravenously show decreased activation of astrocytes and microglia in the spinal cord, reduced pathological protein buildup in the spinal cord, and increased anti-oxidant protein production and activity. MSC treated mice also have less endogenous glutamate release, a pathological finding seen in patients with ALS. Each of these cellular changes could contribute to improved clinical outcomes for patients with ALS.^145^ Adipose-derived MSCs administered intravenously to ALS murine models delay symptom onset and motor deterioration. This treatment also improves lumbar motor neuron survival, possibly via upregulation of glial-derived neurotrophic factor and basic fibroblast growth factor.^146^ Thus, intravenous administration of hUCB cells or MSCs can improve symptomology and be neuroprotective in ALS animal models, encouraging future clinical applications of intravenous stem cell injection. Ultimately, stem cells offer enormous potential to reduce ALS pathology. Considering the ample evidence for ALS as a neurodegenerative disease with neurovascular disorder, it is of immense clinical relevance that many of these preclinical studies on cell-based therapies demonstrate restored BCNSB integrity.

## Clinical Applications of Stem Cells in ALS

Building on exciting findings in animal models, clinical applications of stem cells have been employed for ALS, however, none have focused primarily on BCNSB strengthening and restoration (Table 1). Some theories of MSC benefit in ALS include MSCs becoming the vascular endothelium to restore microcirculation to damaged neuromuscular junctions.^147,148^ Other theories, however, propose that MSCs exert their therapeutic effects by stimulating neurogenesis, releasing NTFs, and modulating inflammatory responses.^147-152^ This immunomodulatory effect of MSCs is vital to their role in ALS therapeutics given the role of microglia in motor neuronal death and ALS symptoms.^153^ Intravenous administration of MSCs may also modulate local inflammation via lymphocyte proliferation, as cell administration increases Treg cells and decreases inflammatory lymphocyte propagators such as CD40(+), CD83(+), CD86(+), and HLA-DR on myeloid dendritic cells.^154^ Intravenous administration is an important focus of future study, as it is less invasive and may have less side effects. Comparing intrathecal to intravenous administration, however, shows that both are safe methods for BM-MSC administration.^155^ Further analysis of the optimal route of administration considering effectiveness, cell viability, cell migration, and invasiveness are needed. Intrathecal bone marrow MSCs also shifts microglial differentiation, increases Tregs, and amplifies anti-inflammatory cytokines, including IL-4, IL-10, and TGF-β.^156^ TGF-β release may offer even further benefit, as it can reduce MCP-1 levels. MCP-1 is increased in ALS CSF samples and high glial cell MCP-1 concentrations are positively correlated with accelerated disease progression.^22,157,158^ Unfortunately, most trials can only report safe administration with weak symptom relief.^154,159-163^ This has not perturbed researchers, however, and various methods and stem cell variations continue to be tested to optimize cell-based treatments for ALS.

**Table 1.** Recent updates on stem cell trials in ALS.

Trial	Sample, *N*	Cell type	Route	Results as available	Status
Effect of intrathecal administration of hematopoietic stem cells in patients with amyotrophic lateral sclerosis (ALS) NCT0193332	14	Hematopoietic stem cells	Intrathecal		Completed as of April 2015
Intravenous transplantation of mesenchymal stem cell in patients with ALS NCT01759797	6	Bone marrow derived mesenchymal stem cell (BMSC)	Intravenous		Completed as of March 2014
Intravenous injection of adipose-derived mesenchymal stem cell for ALS NCT02492516	19	Adipose derived mesenchymal stem cell	Intravenous		Completed as of April 2017
Repeated mesenchymal stem cell injections in ALS^164^ NCT04821479	20	Mesenchymal stem cells (MSC)	Intrathecal	No serious adverse events with clinical benefits related to the intervals between cell administration	Completed as of March 2021
Human neural stem cell transplantation in amyotrophic lateral sclerosis (ALS) (hNSCALS) NCT01640067	18	Human neural stem cells	Intra-spinal cord		Completed as of December 2015
Intrathecal transplantation of mesenchymal stem cell in patients with ALS NCT01771640	8	BMMSCs	Intrathecal		Completed as of December 2014
The evaluation of the effect of mesenchymal stem cells on the immune system of patients with ALS (ALSTEM) NCT04651855	20	MSCs	Intrathecal		Active, not recruiting as of May 2022
Mesenchymal stem cells for treatment of amyotrophic lateral sclerosis (ALS) NCT01142856	1	MSCs	Intraspinal		Completed as of April 2011
Safety of cultured allogeneic adult umbilical cord derived mesenchymal stem cell intrathecal injection for ALS NCT05003921	20	Allogeneic adult umbilical cord derived mesenchymal stem cells	Intrathecal		Recruiting
Compassionate treatment: an exploratory clinical trial to assess treatment of amyotrophic lateral sclerosis NCT02383654	1	Autologous adipose-tissue derived stem cells	brain transplantation of ADSCs and combines intravenous infusion ADSCs		Completed as of January 2016
CNS10-NPC-GDNF for the treatment of ALS NCT02943850	18	Human neural progenitor cellsexpressing GDNF (CNS10-NPC-GDNF)	Intraspinal		Completed as of October 2019
Clinical Trial on the use of autologous bone marrow stem cells in amyotrophic lateral sclerosis (extension CMN/ELA) NCT01254539	63	Bone marrow stem cells(BMSC)	Intratheacal		Completed as of November 2015
A dose-escalation safety trial for intrathecal autologous mesenchymal stem cell therapy in amyotrophic lateral sclerosis NCT01609283	27	MSCs	Intrathecal		Completed as of January 2019
Clinical Trial on the use of autologous bone marrow stem cells in amyotrophic lateral sclerosis (CMN/ELA)^158^ NCT00855400	11	BMSCs	Intraspinal	Safe and promotes neurotrophic activity around motor neurons	Completed as of February 2010
Safety and Efficacy Study of Autologous Bone Marrow Derived Stem Cell Treatment in Amyotrophic Lateral Sclerosis^163^ NCT01363401	72	BMSCs	Intrathecal	BM-MSC intrathecal injections are safe and decreases proinflammatory cytokines, will increasing anti-inflammatory cytokines.	Completed as of August 2013
Escalated Application of Mesenchymal Stem Cells in Amyotrophic Lateral Sclerosis Patients NCT02987413	3	MSCs	Intrathecal		Completed as of April 2017
Safety Study of HLA-haplo Matched Allogenic Bone Marrow Derived Stem Cell Treatment in Amyotrophic Lateral Sclerosis NCT01758510	6	HLA-haplo matched Allogenic BMSCs	Intrathecal		Completed as of April 2017
Safety and Efficacy of Repeated Administrations of NurOwn in ALS Patients NCT03280056	263	MSC-Neurotrophic factor (NTF) cells	Intrathecal		Completed as of October 2020
CNS10-NPC-GDNF Delivered to the Motor Cortex for ALS NCT05306457	16	CNS10-NPC-GDNF	Delivered to motor cortex		Recruiting
A Multicenter Phase I/II Clinical Trial to Evaluate Safety of Mesenchymal Stem Cell in Patients With Amyotrophic Sclerosis Lateral NCT02290886	52	MSCs	Intravenous		Completed as of March 2022
Phase 2, Randomized, Double Blind, Placebo Controlled Multicenter Study of Autologous MSC-NTF Cells in Patients With ALS (NurOwn)^154^ NCT02017912	48	Autologous MSC-NTF cells	Combined intramuscular and intrathecal	Safe and improved Revised ALS Functional Rating Scale scores, increased neurotrophic factors and decreased inflammatory markers.	Completed as of July 2016
Intrathecal Autologous Adipose-derived Mesenchymal Stromal Cells for Amyotrophic Lateral Sclerosis (ALS) NCT03268603	60	Adipose-derived MSCs	Intrathecal		Recruiting
Neurologic Stem Cell Treatment Study (NEST) NCT02795052		BMSCs	Intravenous or intranasal		Recruiting
Evaluation the Efficacy and Safety of Mutiple Lenzumestrocel (Neuronata-R Inj.) Treatment in Patients With ALS (ALSummit) NCT04745299	101	Autologous BMSCs	Intrathecal		Recruiting
Autologous multipotent mesenchymal stromal cells in the treatment of amyotrophic lateral sclerosis (AMSC-ALS-001) NCT03828123	26	MSCs	Intrathecal		Completed as of august 2017
Safety study of VM202 to Treat Amyotrophic Lateral Sclerosis NCT02039401	18	VM202 (hepatocyte growth factor containing DNA plasmid)	NA	Safe and slows disease progression	Completed as of December 2017
G-CSF Treatment for Amyotrophic Lateral Sclerosis: A RCT Study Assessing Clinical Response NCT00397423	40	Granulocyte-colony stimulating factor (G-CSF) which stimulates stem cell production in the brain	NA		Completed as of August 2007
The Effect of GCSF in the Treatment of ALS Patients NCT01825551	40	G-CSF which stimulates stem cell production in the brain	NA	G-CSF may accelerate ALS progression in female patients	Completed as of November 2013
Autologous mesenchymal bone marrow stromal cells secreting neurotrophic factors (MSC-NTF), in Patients With Amyotrophic Lateral Sclerosis (ALS)^152^ NCT01777646 & NCT01051882	14	Mesenchymal bone marrowstromal cells secreting neurotrophic factors (MSC-NTF)	Intramuscular and intrathecal	Safe and improve forced vital capacity and of the ALS Functional Rating Scale-Revised score	Completed as of September 2015
Mesenchymal stem cell transplantation in amyotrophic lateral sclerosis: A Phase I clinical trial^147^	10	MSCs	Intrathecal	Safe	
Mesenchymal stromal cell transplantation in amyotrophic lateral sclerosis: a long-term safety study^148^	19	MSCs	Intrathecal	Safe for up to 9 years	
Safety and immunological effects of mesenchymal stem cell transplantation in patients with multiple sclerosis and amyotrophic lateral sclerosis^142^ NCT00781872	19	MSCs	Intrathecal	Safe and stable ALSFRS, increased T-reg cells and decreased lymphocyte and pro-inflammatory cell receptor expression	
Intraspinal neural stem cell transplantation in amyotrophic lateral sclerosis: phase 1 trial outcomes^146^	12	NSCs	Intraspinal	Cervical injection is safe and well-tolerated	
Lumbar intraspinal injection of neural stem cells in patients with amyotrophic lateral sclerosis: results of a phase I trial in 12 patients^149^	12	NSCs	Intraspinal	Safe to implant SCs using surgical procedure, the introduction of stem cells into the spinal cord, and the use of immunosuppressant drugs	
Transplantation of spinal cord-derived neural stem cells for ALS: analysis of phases I and II trials^150^ NCT 01730716	15	NSCs	Intraspinal	Intraspinal, high-dose injections of NSCs are safe at lumbar and cervical levels. Significant functional improvements or disease slowing were not observed.	

Various attempts to strengthen the clinical impact of SCs for ALS have been implemented in clinical trials. Comparing various administration methods, MSCs expressing NTFs were injected intramuscularly (IM) and intrathecally (IT). Intrathecal administration of SCs is widely practiced in ALS trials due to the more immediate contact with the CNS.^164,165^ While direct implantation into the brain or spinal cord may seem even more beneficial, the pathological degradation of long, established MN connections in ALS mitigates the impact of new central motor neuron growth. Furthermore, new motor neurons may have equal risk of adopting ALS pathology due to local environmental factors.^166^ Rather than growing nascent motor neurons, it is more therapeutically beneficial to protect the well-established and educated existing neurons via intrathecal injections.^166^ Some participants received IM and IT injections at separate times, while others received both concurrently. Considering all groups, 50% of patients showed at least 25% reduction in disease progression over time. This was measured by measuring patients’ Amyotrophic Lateral Sclerosis Functional Rating Scale (ALSFRS) scores and functional residual capacities.^165^ The enhancement of NGF in MSC administration may have provided marked benefit, as NTF are neuroprotective. GDNF, brain derived NTF, VEGF, and hepatocyte growth factor were all substantially elevated. These effects are primarily noticeable when GDNF is over-expressed at the muscle level, prompting greater examination of IM injections with GDNF enhanced cells. NurOwn cells are also NTF-enhanced and demonstrate rapid improvement after administration, however followed by a steady decrease in therapeutic potential.^167^ Thus, repeated administration may be beneficial. These cells increase NTF while decreasing inflammation and MCP-1, which correlates to improved ALSFRS-Revised slope. Additionally, miR-132-sp is increased. This miRNA is notable for promoting neuronal growth and vascular strength in the brain and may be another potential target for amplifying stem cell effects in ALS.^168^ Another target miRNA is miR-125b, which, when inhibited, amplifies anti-inflammatory microglial proliferation and NTFs such as BDNF.^169,170^ Neurotrophic bone marrow cellular nests are also shown to reduce MN degeneration and death and stabilize functional residual capacities and ALSFRS scores.^171^ Another stem cell adjustment may involve Gi signaling pathways. MN firing has been shown to restore BSCB integrity by activating Gi cascades in astrocytes, eventually leading to Wnt7a and Wnt5a transcription.^172^ These genes promote BBB development and maintenance in utero and throughout life.^173-175^ Thus, enhanced Gi signaling or increased Wnt expression in administered stem cells may also amplify their potential for ALS treatments. The demonstrated safety of stem cells, along with many initiatives to enhance cell-based therapy effectiveness, offer hope for a promising stem cell treatment for ALS in the near future.

In addition to various cell enhancements, such as NTFs, and optimizing cell administration protocols, modifying cell dosage can also enhance SC function. Administering 2 injections 26 days apart significantly improved (by 50%) the ALSFRS-R slope, demonstrating delayed disease progression compared to control groups with no cell treatments. Survival and tracheostomy-free survival times were predicted to be longer in younger MSC treated patients. Thus, earlier cell treatments may provide substantial therapeutic benefit.^176^ After longer follow-up, however, these benefits were not noticeable, suggesting continuous treatments may be warranted. Another study injected 4 rounds of MSCs in ALS patients, demonstrating continuous symptom improvement in the majority of patients after each subsequent injection. Overall, 13 patients had a >25% improvement in ALSFRS-R slope and 36.8% of patients showed significant clinical improvement.^177^ The administration of SCs overtime showed promising results, and future studies may also examine the therapeutic benefit of stem cells administered to multiple spinal levels, which was very beneficial in a MSC-treated rat model of ALS.^178^ Given the multitude of promising results in preclinical stem cell studies for ALS, and the ubiquitous safety reports clinically, future initiatives should aim to enhance the therapeutic benefits of cell-based treatments. Whether this involves NTF enhancement, different injection sites, or repeated administration, novel approaches must be taken to exhume SCs potential to treat a deadly and untreatable disease. Furthermore, future studies must focus on the application of stem cells to reduce BCNSB damage, as early reversal of this permeability may ameliorate ALS progression and significantly improve prognosis.

## Conclusion

There are an exorbitant number of studies highlighting BCNSB disruption in ALS pathology. Given the incredibly scarce and moderately effective therapies available, it is imperative to expand treatment targets (i.e., neurovasculature) and explore novel therapeutic tools, such as stem cells. As ECs regress, pericytes are destroyed, tight junctions become diminished, and MMPs destroy the supportive basement membrane of the NVU. Injecting stem cells may be a robust approach to combat NVU impairment towards restoration of CNS homeostasis. Considering the two theories on the mechanism of MSC effectiveness in ALS (i.e. acting as vascular endothelial cells to restore the BCNSB or releasing neuroprotective and anti-inflammatory signals), it is important for future research to also elucidate the underlying mechanisms of stem cells in ALS. A better understanding of MSCs’ mechanisms in ALS may allow for more powerful clinical applications of stem cells. With further neurovasculature-directed research, stem cells aimed to restore the BBB and BSCB in patients with ALS may be a revolutionary treatment for these patients’ with currently poor prognoses.

## Data Availability

Data sharing is not applicable to this article as no new data were created or analyzed in this study.
